# Spatial Differentiation of Physical and Chemical Soil Parameters under Integrated, Organic, and Biodynamic Viticulture

**DOI:** 10.3390/plants9101361

**Published:** 2020-10-14

**Authors:** Maximilian Hendgen, Johanna Döring, Verena Stöhrer, Fabian Schulze, Ruth Lehnart, Randolf Kauer

**Affiliations:** 1 Department of Soil Science and Plant Nutrition, Hochschule Geisenheim University, Geisenheim, D–65366 Hessen, Germany; Ruth.Lehnart@hs-gm.de; 2 Department of General and Organic Viticulture, Hochschule Geisenheim University, Geisenheim, D–65366 Hessen, Germany; Johanna.Doering@hs-gm.de (J.D.); VerenaSina.Leser@mail.hs-gm.de (V.S.); Fabian.Schulze@mail.hs-gm.de (F.S.); Randolf.Kauer@hs-gm.de (R.K.)

**Keywords:** bulk density, copper, management, SOC, vineyard

## Abstract

Vineyard soils show an increased risk of degradation due to being intensively cultivated. The preservation of soil integrity and fertility is a key concept of organic and biodynamic farming. However, both systems are also subject to criticism due to their higher amount of plant protection products used and their increased traffic intensity compared to integrated viticulture, both detrimental to soil quality. The aim of this study was therefore to assess long-term effects of these three management systems on chemical and physical soil quality parameters. For this purpose, topsoil samples were taken in a long-term field trial vineyard at different positions and examined for bulk density, available water capacity (AWC), soil organic carbon (SOC), N, pH, and for total and bioavailable copper (Cu) concentrations. Biodynamic plots had a lower bulk density and higher SOC concentration than the integrated ones, which is probably due to the species-rich cover crop mixture used in the inter-row. However, organic and biodynamic farming showed an accumulation of copper in the under-vine area and in the tractor track, which is problematic for soil fertility in the long-term. Therefore, alternatives for copper in plant protection are necessary to ensure sustainable soil quality through organic and biodynamic viticulture.

## 1. Introduction

The quality of agricultural soils is mainly measured by their productivity, i.e., the yield and nutritional value of the crops grown on them [1]. This productivity depends on the physical, chemical, and biological soil parameters and their various interactions [2]. Since soil biology is difficult to record, and its effect on plant production has only partially been unraveled by now, the chemical and physical parameters have traditionally been given greater importance in soil evaluation [2]. Among the most important chemical soil parameters are the pH value, the mineral and organic composition of soils. Physically important parameters are the particle size distribution, the bulk density, and the pore structure, which in interaction are decisive for the air and water balance of the soil [1].

However, soil quality—just like the parameters that determine it—is not a static condition, but is influenced and changed by land use and management [1]. In agricultural soils, erosion, nutrient losses, decline in organic matter, compaction, and contamination inputs are the main threats that can lead to a loss of soil quality [3]. The soils of permanent crops, such as grapes are particularly at risk of a reduction in quality, as they are degraded more than arable or grassland areas due to intensive cultivation and the comparatively high use of plant protection products [4,5]. At the same time, the long-term productivity of such crops depends crucially on the permanent safeguarding of soil quality [6,7].

Viticulture in temperate growing regions is usually carried out as a spatial culture in the form of rows, with the inter-row space serving as tramlines for mechanical cultivation with tractors [8]. This results in a high risk of compaction for the lane due to the frequent passes with heavy machines [9]. An attempt is made to counteract this danger by greening the inter-row space. In contrast, the under-vine area is usually kept free of vegetation in order to ensure good aeration of the grape zone, which results in highly differentiated soil management on a small scale.

The most widespread management system in viticulture is integrated cultivation, which is based on a combination of biological, biotechnical, plant breeding, and cultivation techniques [10]. Over the past 15 years, there has been an increasing trend towards organic or biodynamic viticulture. In the European Union, 340,000 hectares (8.7% of the total area under vines) are currently managed organically (status 2018) [11]. Organic farming systems differ from integrated viticulture in the use of copper (Cu), sulfur, and plant strengtheners instead of synthetic fungicides, in the use of organic instead of mineral fertilizers, and in the absence of chemical herbicides [12,13]. Furthermore, the use of species-rich cover crop mixtures is intended to increase biodiversity and promote soil fertility by building up organic matter. Biodynamic viticulture also follows an anthroposophical approach, in which additional plant and animal preparations are used [14].

On the one hand, organic farming systems, which aim to achieve a more environmentally friendly and sustainable production by exclusively using products of natural origin, have been shown to have increased soil microbial activity and a modified community composition, compared to integrated cultivation [15,16,17]. The positive effects of organic and biodynamic viticulture on soil microbial properties are reported to increase over time since conversion [18].

On the other hand, organic viticulture is also criticized for its use of Cu with regard to soil protection. As a heavy metal, Cu is not degraded or leached off the soil, but is predominantly bound to organic matter and clay minerals and thereby immobilized [19]. Regular application leads to an accumulation in the topsoil [20]. As a result of immobilization, the bioavailability and thus the toxicity of Cu in the soil is reduced [21]. Therefore, in addition to the total Cu concentration, the amount of bioavailable Cu is of great ecological and agronomic importance [22,23]. The bioavailability of Cu highly depends on the distribution of Cu among soil components and their ability to adsorb or desorb Cu on one hand, and chemical soil parameters, such as the content and nature of organic matter, clay minerals, metal oxides, cation exchange capacity, redox potential, and pH [23]. In order to determine the bioavailability of copper, weak chemical extraction agents are used to simulate the extraction capacity of roots. Common extraction agents for this purpose are ethylenediaminetetraacetic acid (EDTA), diethylenetriaminepentaacetic acid (DPTA), calcium chloride (CaCl_2_) or a calcium chloride/DPTA mixture (CAT). To reduce the toxic effect of high Cu concentrations on soil organisms and plants, there is a legal upper limit for the use of Cu in plant protection in the European Union [24,25,26]. In organic viticulture, the use of Cu in plant protection against downy mildew (*Plasmopara viticola*) is restricted to 6 kg ha^−1^ a^−1^ [13]. Beyond this, Cu application in viticulture in Germany is limited to 3 kg ha^−1^ a^−1^ due to the application instructions in the registration reports of copper-containing fungicides [27,28]. The amounts of Cu used in plant protection are generally higher in organic compared to integrated management, since the latter mostly relies on synthetic fungicides. Nonetheless, several studies comparing Cu contents in soils of organically and conventionally managed vineyards did not detect differences among the systems [18,29,30,31]. This might be because Cu contents in vineyard soils have accumulated over the past century, thereby, masking recent management effects [18,30,32]. On the other hand, Cu accumulation in the soil is a gradual process and might only be detected in the long-term. One study comparing organic and conventional management over nine years in Italy detected higher Cu contents in organically managed soils underlining the necessity of long-term field trials assessing soil quality in organic viticulture [33].

The lower effectiveness of the pesticides used in organic viticulture, as well as the under-vine weed control by mechanical means also requires a higher number of tractor passes, exposing the tractor track to a higher load than in integrated viticulture [9]. Compacted soils are difficult to root through and have a poor water and gas balance [34].

Although, there are numerous publications on individual effects of Cu, herbicide, or fertilizer application on soil quality parameters, holistic assessments regarding the effects of management systems on physical and chemical soil parameters are still rare. Such assessments are hampered by the fact that effects such as Cu accumulation, soil compaction or the build-up of organic matter only develop in the long-term [26,35].

Several studies comparing organic and conventional viticulture found contents of soil organic carbon (SOC) and total N not to be different [18,36,37]. Gutiérrez-Gamboa et al. [38], in contrast, found N content in organically managed soils in Chile to be higher compared to integrated and conventional management, respectively. Researchers in south of France found a long-term increase in organic carbon and nitrogen due to organic farming, but no increase in bioavailable Cu [29]. Characteristic effects of organic and biodynamic viticulture are higher cumulative soil respiration, higher content of microbial biomass C, and a higher ratio of microbial biomass C to organic C indicating higher soil microbial activity [18,29,37,39,40,41]. In regards to soil physics, Coll et al. [29] reported a higher bulk density and thus increased soil compaction, as a result of organic farming. However, their experimental design consisted of various plots managed by different owners, and only inter-row soil was sampled. Furthermore, it is to be expected that positive or negative treatment effects will not show up over the whole area, but spatially differentiated due to the row-shaped vineyard design. Such spatial variability has repeatedly been reported for soil compaction in vineyard soils [9,35,42], and a comparable zoning can be expected for Cu accumulation, due to drip losses and leaf fall.

The aim of this study was therefore to assess spatial long-term effects of the different management systems in viticulture on physical and chemical soil quality parameters. For this purpose, bulk density and plant-available water capacity (AWC), soil organic carbon (SOC) and nitrogen (N), as well as the concentrations of total and bioavailable Cu were recorded, spatially separated into under-vine area, tractor track, and the middle of the greened inter-row within a unique long-term field experimental vineyard. The results provide an insight into the underground variability in permanent crops and improve the data basis for a thorough evaluation of integrated, organic, and biodynamic viticulture.

## 2. Results

Analyses of variance revealed a significant effect of the management system as well as of the row position on mean bulk density (Table 1). Mean bulk density was highest in the soil of integrated plots (1.53 g/cm^3^) and differed significantly from biodynamic management (contrast *p* = 1.46 × 10^−2^), which had the lowest mean bulk density (1.47 g/cm^3^). The organically managed soil ranged between them (mean 1.51 g/cm^3^) without a statistical difference. Bulk density varied significantly among all three positions. The highest soil compaction was found in the tractor track (mean 1.57 g/cm^3^), followed by the middle of the greened inter-row (mean 1.51 g/cm^3^). The under-vine area showed the lowest bulk density (1.41 g/cm^3^ in average). None of the tested physical or chemical soil parameters showed a significant interaction effect among management systems or positions (Table 1).

The amount of plant-available water was affected solely by the position and did not differ among management systems (Table 1). The soil of the greened inter-row had a significantly higher available water capacity (AWC) (16.4% on average) compared to both the under-vine area (*p* = 0.012) as well as the tractor track (*p* = 0.044). Under-vine area and tractor track were almost 2% lower in AWC and did not differ from each other (*p* = 0.668).

The pH of the soil samples ranged between 7.12 and 7.23 on average and no difference was found between the different treatments or positions. (Table 1).

Soil N did not differ neither among management systems nor among positions. Mean values ranged between 0.11% and 0.13% (Table 1).

The amount of soil organic carbon (SOC) was affected by the management system, but not by the position within the vineyard (Table 1). SOC was shown to be significantly higher in the biodynamically managed plots (mean 1.41%) compared to both the integrated (mean 1.09%) and organic management (mean 1.15%). No statistical difference was detectable between the integrated and the organic system. In the inter-row SOC was slightly higher (mean 1.35%) compared to the under-vine area (mean 1.13%) and the tractor track (mean 1.17%), but results were not significant.

The C/N ratio did not show neither a significant position effect nor a significant management effect. However, as a consequence of the results on N and SOC, the biodynamic treatment (12.31) tended to have a slightly higher C/N ratio than the organic (10.2) and the integrated system (10.15) (Table 1).

The total Cu concentration in the soil samples ranged from 30.6 to 119.8 mg/kg soil, but only four samples had a value greater than 100 mg/kg. Analyses of variance revealed a significant effect of the management system as well as of the alley position on the total and bioavailable Cu concentration (Table 1). The concentrations of total, as well as of bioavailable copper were significantly higher in the soils of the organically and biodynamically managed plots compared to the integrated management. Mean values of total Cu were 74, 87, and 87 mg/kg soil and mean values of bioavailable Cu were 19, 25, and 22 mg/kg soil for the integrated, organic, and biodynamic management, respectively. The ratio of bioavailable to total Cu in soil was similar in all management systems and ranged between 25 and 28%. In relation to the position, the concentrations of both total and bioavailable Cu were significantly increased in soils of the under-vine area (Table 1). Mean values of total Cu were 88.5, 78.5, and 81.8 mg/kg soil for the under-vine, tractor track, and inter-row position, respectively. The mean values for bioavailable Cu per position amounted to about 26% of the total Cu values. Concentrations of total, as well as bioavailable Cu did not differ between tractor track and inter-row space.

### Spatial Differentiation of Management Effects

Soil physical and chemical parameters, which showed a significant management effect (as was the case for bulk density, SOC, and total and bioavailable Cu), were further examined on spatially differentiated scale. The aim of the differentiation was to localize more precisely positive and negative impacts of long-term integrated, organic, and biodynamic viticulture on the soil. This is important to provide guidance for the different management systems.

**Figure 1:**
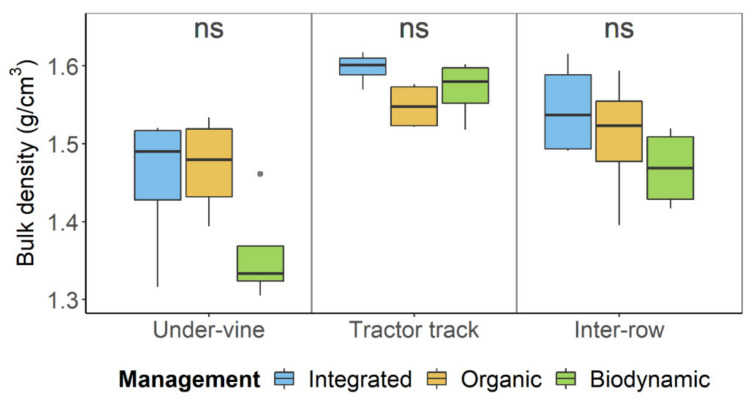
Bulk density of vineyard soil under integrated, organic and biodynamic management, separated by the row positions under-vine area, tractor track and middle of the greened inter-row.

Bulk density under integrated management was significantly higher compared to biodynamic management (Table 1). In relation to the spatial variability within the management systems, bulk density was lowest in the under-vine area and highest in the tractor track for all three management systems (Figure 1). Integrated management showed the highest mean density in the tractor track and the inter-row, and the median was highest at all three positions. In contrast, biodynamic farming, which in average had a significantly lower bulk density compared to integrated farming across all three positions, tended to have a lower bulk density in the under-vine area and the inter-row space. Nevertheless, none of the three examined positions showed a significant management effect in their individual consideration.

**Figure 2:**
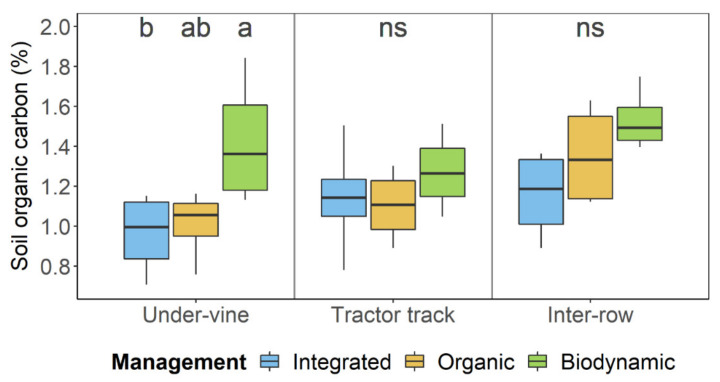
Soil organic carbon of vineyard soil under integrated, organic and biodynamic management, separated by the row positions under-vine area, tractor track and middle of the greened inter-row. Management systems with different letters on top differ significantly with α = 0.05 according to least significant difference test (*n* = 4).

Soil organic carbon was significantly higher in soils of biodynamic management and tended to show higher values in the inter-row space compared to the under-vine area (Table 1). In the under-vine area, SOC was highest for the biodynamic treatment and differed significantly from the integrated treatment (Figure 2). Moreover, the biodynamic treatment showed the highest SOC values in all three positions within the vineyard.

**Figure 3:**
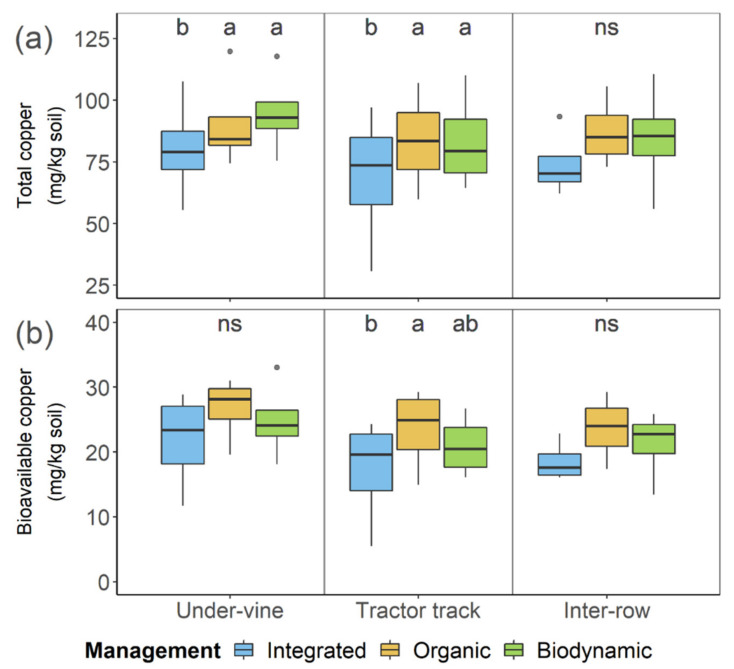
Total Cu (**a**) and bioavailable Cu (**b**) of vineyard soil under integrated, organic and biodynamic management, separated by the row positions under-vine area, tractor track and middle of the greened inter-row. Management systems with different letters on top differ significantly with α = 0.05 according to least significant difference test (*n* = 4).

The generally increased total Cu concentrations in the soil of the organic and biodynamic management system thereby showed to base on significantly higher Cu levels in the under-vine area (*p* = 5.2 × 10^−3^) and in the tractor track (*p* = 0.039) (Figure 3A). Mean total Cu concentrations of the organic and the biodynamic treatment exceeded the integrated average value by 10 and 15 mg per kg soil in the under-vine area, and by 13 mg per kg soil in the tractor track. By contrast, no difference among the management systems was detected for the inter-row position (*p* = 0.133), although the total Cu level of the organic and biodynamic samples also tended to be slightly higher than the one of the integrated soil.

Even though the bioavailable Cu concentration—averaged across all management systems—was highest in the under-vine soil, the spatially separated results solely showed a significant differentiation among the management systems in the tractor track (*p* = 0.043) (Figure 3B). The mean bioavailable Cu values of the organic and biodynamic soils also tended to be higher in the under-vine area as well as the inter-row section, but treatments did not differ significantly (*p* = 0.119 | *p* = 0.088).

## 3. Discussion

Repeated tractor passes in the experimental vineyard led to considerable soil compaction in the tractor track. Therefore, our results are basically in line with the reports of various other research groups who also found compaction in the wheel track of tractors in agricultural soils [34,35,42,43]. In comparison of the farming systems, however, organic and biodynamic farming did not lead to higher soil compaction, although both systems had an average of one more tractor pass per year and row compared to the integrated treatment for the years 2006 to 2018. Rather, the soil of the biodynamic management even had a lower bulk density than the one of the integrated treatment, which is contrary to the findings of Coll et al. [29]. The differences in soil bulk density among the treatments were presumably due to the difference in cover crop vegetation. A positive effect of greening on soil structure, which is based on a combination of aboveground cushioning effect of the plant biomass, a soil aggregate stabilization effect by the root system, and a regular input of organic carbon is widely recognized [44,45]. Our results show that a diverse vegetation cover is more effective than sward in preventing soil compaction. This is demonstrated firstly by the results of an evaluation of the cover crop vegetation in the experimental vineyard in 2019, in which plant diversity, maximum height, and percent coverage of the soil of the cover crop biomass were recorded at three dates during the growing season (June, July, August) (Table S1). While no difference in the coverage of the soil was found, the maximum growth height of the cover crop biomass was significantly higher (*p* = 4.86 × 10^−4^) in the organically (mean 32.0 cm) and biodynamically (mean 24.3 cm) managed plots compared to the integrated management (mean 12.6 cm), giving them a higher cushioning ability. Furthermore, diverse cover crop mixtures usually have a denser and deeper root system than grass, thereby increasing their soil-stabilizing effect [46]. In addition, a larger plant biomass and a more intensive root system lead to a higher input of organic carbon into the soil. In agreement with this, the organic and biodynamic treatment tended to show higher SOC values in the inter-row area than the integrated treatment. This is in accordance with findings of Coll et al. [29]. Averaged over all positions, SOC was significantly higher in the biodynamic compared to the integrated treatment. Taken together, the use of species-rich cover crop mixtures can effectively counteract the risk of soil compaction due to frequent tractor passes in organic and biodynamic viticulture.

The significantly higher SOC concentration of the biodynamic treatment was—as already mentioned—presumably caused by its cover crop mixture. In the 2019 cover crop evaluation, the biodynamically managed plots did not only show an increased maximum plant height compared to sward in the integrated treatment, as did the organic plots, but also showed a significantly higher number of plant species (Figure S1), compared to the organic and integrated treatment (*p* = 8.21 × 10^−5^). The average number of species within the cover crop in the biodynamic plots was 13.3 compared to 6.3 in the integrated, and 9.5 in the organic plots, respectively. Plant species differ in terms of their carbon input to the soil due to their shoot/root ratio and their rooting depth, and a positive correlation has been found between plant functional diversity and SOC concentration [47,48,49]. However, in terms of spatial differentiation analysis, the higher SOC level of the biodynamic treatment was most evident in the under-vine area. The mechanical under-vine cultivation of the organic and biodynamic treatment presumably left more vegetation than the glyphosate treatment of the integrated plots, resulting in a comparatively higher input of organic carbon in these treatments. It should be noted, however, that the biodynamic treatment even showed more SOC than the organic treatment, although they did not differ in terms of cover crop mixture and under-vine management. Biodynamic preparations, especially horn manure and compost preparations, may be involved in enhancing root growth and biomass development of cover crops sown [50], but the mode of action still remains unclear.

Soil compaction in the lane is presumably also responsible for the reduced AWC of the soil within the lane zone, as compacted soils possess less pore volume [51]. Although the under-vine areas of all three management systems were characterized by the lowest bulk density, its AWC only corresponded to that of the rather compacted tractor track. Therefore, it can be assumed that the low bulk density is mainly due to a high proportion of coarse pores, which play virtually no role in the water supply of the plant. The inter-row samples on the other hand tended to have a higher AWC, indicating a higher proportion of medium pores that are important for the water storage capacity of soils.

In general, the fertility and productivity of agricultural soils strongly correlates with their SOC concentration [52,53]. SOC promotes the formation of clay-organic matter complexes and thereby aggregate stability in the soil [54]. Plant nutrients are released during the mineralization of organic matter, and humic substances can reversibly adsorb nutrients [51]. Organic matter also possesses a high water holding capacity [51], which apart from the better soil structure, can provide an explanation for the generally higher AWC in the inter-row zone of all treatments. This result is in line with that of a long-term farming comparison of arable soils [55]. In addition, SOC as the primary food of soil microorganisms affects the microbial community composition and activity, and corresponding differences among management systems have already been demonstrated in the same experimental vineyard as well as in other studies [15,16,55,56]. It should be noted that management effects on the SOC balance of soils in field experiments can be expected after five years at the earliest [57], which underlines the importance of long-term experiments like the current one.

In contrast to the differences in SOC concentration, no difference was found for N among the systems. The reason for this is probably the balanced experimental design with regard to nutrient applications in the three systems and the overall low N inputs by fertilizer applications. Mineralized N contents in the soil in the same trial after conversion revealed higher values for organic, and biodynamic management, respectively. Yeast-available N in ripe wine grapes and in juices from the same trial also showed higher values in the organic and the biodynamic treatments [58].

Although management systems differed in SOC in the current trial, no difference in the C/N ratio was detected among the systems. The biodynamic treatment having a significantly higher SOC content showed a slightly higher C/N ratio compared to organic and integrated management, but the balanced experimental setup did not lead to major differences in the C/N ratio.

The elevated total and bioavailable Cu concentrations in the soil of the organic and biodynamic plots reflect the use of Cu in crop protection in these two treatments. However, the integrated plots also showed total Cu values above the normal range for unpolluted soils [59,60]. Although, the total Cu concentrations were in all soil samples below the European Union’s critical limit of 140 mg/kg soil [24], only one sample was below the precautionary value of 40 mg/kg in Germany [61]. Other studies on Cu-contaminated vineyard soils, however, revealed in part even substantially higher Cu contaminations [19,59,62,63]. The average difference of 13 mg/kg total Cu between integrated and organic, or biodynamic plots, respectively, based on a trial period of 13 years, corresponds to an annual increase in total Cu concentration of about 1 mg/kg. This result is in line with the theoretical Cu input into the topsoil by plant protection measures, calculated on the basis of the maximum annual application rate of 3 kg Cu per ha as permitted in Germany [27,28]. The Cu input in the current trial was 2.5 kg ha^−1^ a^−1^ on average.

As the spatial differentiation analysis reveals, the increased total Cu levels were located particularly in the under-vine area and the tractor track, which is also the eaves zone of the grapevine canopy. As Cu is quickly attached to organic matter and clay minerals when entering the soil [64], the accumulation of Cu in these areas may be explained by dripping pesticides and leaf fall residues from the canopy. The spatial variability of Cu accumulation as found in our study may also explain the contradicting results to the study of Coll et al. [29], who did not find a significant increase in the available Cu concentration in the soil of organically farmed vineyards in southern France, but only sampled the middle of the inter-row.

Unlike the case of total Cu, a significant difference in bioavailable Cu among the management systems occurred solely in the tractor track between integrated and organic plots. As the availability of Cu in soil depends on numerous factors like pH, redox potential, cation exchange capacity, organic matter, particle size, and the presence of manganese and iron oxides [65,66,67], the ageing of the deposited Cu may vary among row positions. In relation to the ratio of bioavailable to total copper, the CAL extraction of our study with an average of 26% gave comparable results to the extraction agents EDTA or DPTA used in many other studies [26,63,68].

Taken together, the three forms of vineyard management differ with regard to various physical and chemical soil parameters. In this respect, our study shows a spatial soil differentiation with closely spaced zones of better or worse soil quality, which is responsible to varying degrees for long-term position and management effects. Despite a higher number of tractor passes, organic and biodynamic farming did not lead to a higher soil compaction, rather the opposite was the case due to the positive effects of their cover crop mixture. The biodynamic treatment shows particularly positive effects having the lowest bulk density and the highest SOC concentration together with the highest biodiversity of cover crops in the current study. On the other hand, the organically and biodynamically cultivated vineyard soils show a constant accumulation of Cu, which is problematic for soil fertility in the long term. Consequently, research into alternative plant protection agents should be intensified or the cultivation of disease-tolerant varieties, for whose protection less Cu is needed, should be promoted.

## 4. Materials and Methods

### 4.1. Experimental Site

The field experiment was set up in a vineyard located in Geisenheim, Germany (49°59′22.0″ N; 7°57′00.8″ E). The vineyard was planted in 1991 (*Vitis vinifera* cv. Riesling, clone Gm 198–30, grafted on *Vitis berlandieri* Planch. × *Vitis riparia* Michx. cv. SO4 and *Vitis riparia* Michx. × *Vitis cinerea* Engelm. cv. Börner rootstock, respectively) and was 0.8 ha in size. It had a row spacing of 2 m, a vine spacing of 1.2 m and was trained as a single Guyot in a vertical shoot positioning (VSP) system. Until the end of 2005, the whole vineyard was managed according to the Good Agricultural Practice. Since 2006, the organic and biodynamic vineyard plots were managed according to Regulations (EC) No 834/2007 and (EC) No 889/2008, and according to ECOVIN- and Demeter-Standards, respectively, whereas the integrated management still followed the Good Agricultural Practice.

The experimental site was set up as a complete block design with four field replicates, each of which included the three factor levels of the main effect management system. Each main plot per field replicate and management consisted of 4 rows with 32 vines each. The two outer rows per plot were considered as buffer rows, thus sampling just took place in the inner rows of each plot.

In the organic and biodynamic plots, the Wolff-Mixture^®^ (for the composition, please see Table S3 of Döring et al. [58]) was used as cover crop and a mechanical under-vine management was implemented (Table 2). In the integrated plots, a grass mixture (*Lolium perenne* 20% and *Poa pratensis* 80%) was established as cover crop between the rows, and weeds in the under-vine area were controlled by herbicide application in average twice per year. Sowing of the respective cover crop mixtures in all the treatments occurred on 05/10/2006, 04/04/2007, 04/28/2008, 04/11/2011, 07/18/2014, 10/25/2015, and 05/18/2017 according to seasonal conditions. In all three treatments, every second row was ploughed shortly before bloom. Considerable nitrogen introductions in the organic and the biodynamic treatment due to the ploughing of the legumes-rich Wolff-Mixture were compensated by mineral fertilization of the integrated plots (25 kg N ha^−1^ on 07/06/06, 50 kg N ha^−1^ on 06/26/10, 25 kg N ha^−1^ on 07/05/12, and 25 kg N ha^−1^ on 06/16/14). To control *Erysiphe necator* and *Plasmopara viticola* (powdery and downy mildew), systemic fungicides were used in the integrated plots, and copper, sulfur, and plant strengtheners in the organic and the biodynamic treatments. Both, organic and biodynamic treatments received identical soil and vine management practices except that biodynamic preparations (horn manure, horn silica) were additionally applied to the biodynamic plots. All three treatments received compost in 2006, 2007, and 2016. After analysis of the composts the same amount of nitrogen equivalents were applied to every treatment (50 kg N ha^−1^ on 08/24/06, 25 kg N ha^−1^ on 10/24/07, and 7.5 kg N ha^−1^ on 08/24/16). Green waste compost was used for the integrated plots and farmyard manure originating from organic farming was used for organic and biodynamic treatments, respectively. In addition, the biodynamic compost preparations 502–507 were applied to the compost of the biodynamic treatment. For a brief description of the experimental vineyard and the applied management practices, see Döring et al. [58].

From 2006 until 2018 the organic and the biodynamic plots had 9.44 tractor passes per row and year on average due to soil management, hedging, and plant protection, whereas the integrated plots had 8.33 tractor passes per row and year on average. The average Cu application from 2006 to 2018 in the organic and the biodynamic plots was 2.5 kg ha^−1^ a^−1^.

### 4.2. Soil Sampling

Disturbed soil samples for the copper analysis were taken in July 2018 from topsoil (0 to 30 cm) using a Pürckhauer soil sampler. Undisturbed soil samples for the soil physical analyses were collected in March 2019 within the topsoil as well using stainless steel metal rings (100 cm^3^). The metal rings were hammered into a freshly exposed soil profile with the assistance of a sampling head, then dug out and excessive soil was removed using a sharp knife.

**Figure 4:**
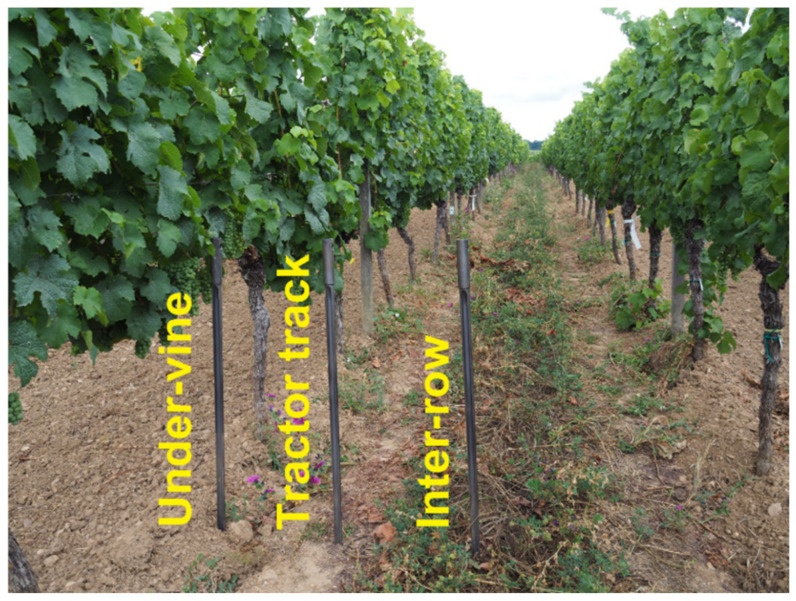
Positioning of disturbed and undisturbed soil sampling between the rows of vines.

Both the disturbed and the undisturbed sampling were carried out at three different positions within each block (Figure 4). The three positions were defined as follows:

- in the under-vine area which has been kept free of vegetation by herbicide or mechanical means
- in the tractor track of the cover cropped inter-row (which was also the draining zone of the canopy)
- in the middle of the cover cropped inter-row between the two main tractor tracks.

The disturbed samples were generated as mixed samples consisting of four drillings per management block × position combination. For the undisturbed samples, one trench covering all three positions was dug out per management block. For each position within a trench, four samples were taken, analyzed separately and finally averaged to ensure representativeness.

### 4.3. Soil Physical Analysis

Soil moisture retention capacity was analyzed using a pressure plate apparatus. First, the undisturbed samples within the metal rings were covered with a parchment paper, saturated with distilled water and then placed on a waterlogged ceramic plate within the pressure vessel. Soil water tension levels were adjusted by pressurizing the vessel, and water retention of the samples at pF 2.5 and pF 4.2 was measured gravimetrically at equilibrium. The plant-available water content (AWC) was calculated as pF 2.5–pF 4.2. Bulk density was calculated as sample dry weight per sample volume (=100 cm^3^).

### 4.4. Chemical Parameter Analysis

Soil total carbon (C) and nitrogen (N) were analyzed according to DUMAS and soil organic carbon (SOC) was determined by subtracting carbonate carbon (determined according to SCHEIBLER) from total carbon.

Total copper was extracted with aqua regia, bioavailable copper was extracted using CAT (c(calcium chloride) = 0.01 M, c(diethylenetriaminepentaacetic acid) = 0.00002 M) and both were measured via ICP-OES. A detailed description of the analytical methods used can be found in Hendgen et al. [15].

### 4.5. Statistics

Statistical analyses were carried out using the R software and the RStudio graphical user interface [69,70]. Analyses of variance were calculated based on a linear mixed model with management and position as fixed and block as random factor. Soil parameters that were significantly affected by the management system or the position were subjected to least significant difference as post-hoc test.

## Figures and Tables

**Table 1:** Effect of management system, alley position and their interaction on the investigated physical and chemical soil parameters.

	*p*_management_	Integrated	Organic	Biodynamic	*p*_position_	Under-Vine	Tractor Track	Inter-Row	*p*_management:position_
Bulk density (g cm^−3^)	0.042 *	1.53 ± 0.09 ^a^	1.51 ± 0.07 ^ab^	1.47 ± 0.1 ^b^	2.40 × 10^−5^ *	1.43 ± 0.09 ^c^	1.57 ± 0.03 ^a^	1.51 ± 0.07 ^b^	0.324
AWC (%)	0.179	15 ± 1.25	14.9 ± 1.68	15.9 ± 1.99	8.88 × 10^−3^ *	14.8 ± 1.4 ^b^	14.6 ± 1.6 ^b^	16.4 ± 1.6 ^a^	0.107
pH	0.257	7.13 ± 0.32	7.23 ± 0.13	7.15 ± 0.08	0.32	7.19 ± 0.22	7.21 ± 0.19	7.12 ± 0.21	0.992
N (%)	0.6	0.11 ± 0.02	0.12 ± 0.05	0.12 ± 0.05	0.357	0.11 ± 0.05	0.11 ± 0.02	0.13 ± 0.02	0.867
SOC (%)	5.23 × 10^−3^ *	1.09 ± 0.24 ^b^	1.15 ± 0.24 ^b^	1.41 ± 0.24 ^a^	0.071	1.13 ± 0.31	1.17 ± 0.22	1.35 ± 0.25	0.564
C/N	0.073	10.15 ± 1.36	10.2 ± 2.39	12.31 ± 2.99	0.853	10.76 ± 3.57	11.22 ± 1.98	10.68 ± 1.73	0.943
Total Cu (mg kg^−1^ soil)	1.52 × 10^−4^ *	74.3 ± 20.4 ^b^	87 ± 16.9 ^a^	87.4 ± 18.9 ^a^	7.01 × 10^−3^ *	88.5 ± 18.9 ^a^	78.5 ± 22.1 ^b^	81.1 ± 16.6 ^b^	0.879
Bioavailable Cu (mg kg^−1^ soil)	1.33 × 10^−3^ *	19.2 ± 6.5 ^b^	24.6 ± 5.3 ^a^	22.3 ± 5.3 ^a^	1.28 × 10^−2^ *	24.5 ± 6.1 ^a^	20.6 ± 6.6 ^b^	21.1 ± 4.7 ^b^	0.992

Analyses of variance were calculated based on linear mixed models. Significant factor effects (with α = 0.05) are marked with a *. Average values ± standard deviation are given per management system and position, respectively, and different superscript letters indicate statistically significant differences among factor levels in case of a significant management or position effect.

**Table 2:** Overview of the general management in the integrated, organic, and biodynamic plots.

	Integrated	Organic	Biodynamic
cover crop	sward (every 2nd row cultivated)	multi-species mixture (every 2nd row cultivated)	
under-vine-management	herbicides	mechanically	
fertilization	green waste compost + mineral fertilizers (according to N_min_ analysis)	farmyard manure + rolling or cultivation of cover crop	farmyard manure with biodynamic preparations (or cow pat pit preparation) + rolling or cultivation of cover crop
plant protection	systemic fungicides botryticides	copper (max. 3 kg/ha and year), wettable sulfur plant resistance improvers	
	mating disruption method against grape berry moth		
biodynamic preparations	-	-	horn manure, horn silica compost preparations

## Supplementary Materials

The following are available online at https://www.mdpi.com/2223-7747/9/10/1361/s1, Table S1: Effects of management system on maximum height of the cover crop biomass and soil coverage [%] in 2019, Figure S1: Biodiversity of cover crops in 2019 expressed as species richness.
