# Supplementary material for: Spatial Differentiation of Physical and Chemical Soil Parameters under Integrated, Organic, and Biodynamic Viticulture
Source: Plants (Basel). 2020 Oct 14;9(10):1361. doi: 10.3390/plants9101361 (PMC7602175; doi:10.3390/plants9101361)
Supplement: Supplementary file 1 [file plants-09-01361-s001.pdf]

Supplementary Information

Table S1. Effects of management system on maximum height of the cover crop biomass and soil coverage [%] in 2019.

	$p_{management}$	Integrated	Organic	Biodynamic
Cover crop height (cm)	**4.86E-04**	12.6 ± 5.1 [b]	32.0 ± 22.0 [a]	24.3 ± 21.4 [a]
Degree of soil coverage (%)	0.053	83.8 ± 16.0	66.3 ± 33.3	65.8 ± 33.4

Maximum height of cover crop biomass and soil coverage in greened inter-row spaces were assessed throughout the growing season 2019 (06/03/2019, 07/10/2019, 08/24/2019) on 1 m^2 per management and field replicate which was chosen randomly.

Analyses of variance were calculated based on linear mixed models. Significant factor effects (with $\alpha = 0.05$) are marked bold. Average values ± standard deviation are given per management system and different superscript letters indicate statistically significant differences among factor levels in case of a significant management effect.

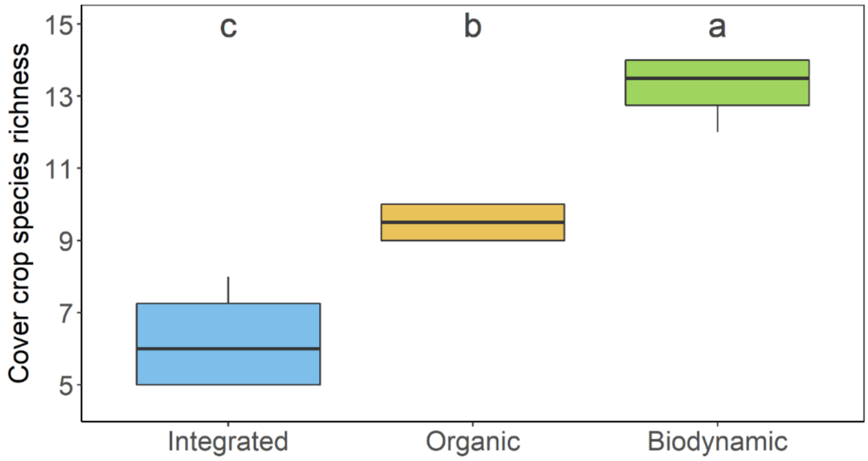

Figure S1. Biodiversity of cover crops in 2019 expressed as species richness.

Cover crop species in greened inter-row spaces were assessed throughout the growing season 2019 (06/03/2019, 07/10/2019, 08/24/2019) on 1 m^2 per field replicate which was randomly chosen. Species were identified according to Aichele and Golte-Bechtle [1], Hanf [2] and Licht [3].

Management systems with different letters on top differ significantly with $\alpha = 0.05$ according to least significant difference test (n = 4).

References

1. Aichele, D.; Golte-Bechtle, M. *Was blüht denn da? Wildwachsende Blütenpflanzen Mitteleuropas*; Franckh`sche Verlagshandlung, W. Keller & Co.: Stuttgart, Germany, 1990; ISBN 9783440037232.
2. Hanf, M. *Farbatlas der Wildkräuter und Unkräuter*; Eugen Ulmer Verlag GmbH & Co.: Stuttgart, Germany, 1998; ISBN 3800140748.
3. Licht, W. *Einführung in die Pflanzenbestimmung. Die wichtigsten Familien und ihre Merkmale*; Quelle & Meyer Verlag: Wiesbaden, Germany, 1995; ISBN 3494012334
